# Imaging Collateral Ventilation in Patients With Advanced Chronic Obstructive Pulmonary Disease: Relative Sensitivity of ^3^He and ^129^Xe MRI

**DOI:** 10.1002/jmri.26273

**Published:** 2018-09-29

**Authors:** Helen Marshall, Guilhem J. Collier, Christopher S. Johns, Ho‐Fung Chan, Graham Norquay, Rod A Lawson, Jim M. Wild

**Affiliations:** ^1^ Academic Unit of Radiology, University of Sheffield Sheffield UK; ^2^ Department of Respiratory Medicine Sheffield Teaching Hospitals NHS Trust Sheffield UK

To the editor:

Endoscopic lung volume reduction (ELVR) can improve lung function, exercise capacity, and quality of life of patients with severe chronic obstructive pulmonary disease (COPD). The assessment of collateral ventilation is key to the success of ELVR, as collateral ventilation from adjacent lung regions prevents collapse of the target lung segment.^1^

The gold standard assessment of collateral ventilation is gas catheter bronchoscopy, but this is an invasive procedure requiring sedation.^1^ Assessment of lobar fissure integrity with anatomical computed tomography (CT) can assist in patient selection,^1^, ^2^ but functional measurements of gas movement within the lungs have direct relevance.

Long‐range diffusion measurements using hyperpolarized ^3^He magnetic resonance imaging (MRI) are sensitive to the effects of collateral ventilation.^3^ Direct imaging of collateral and delayed ventilation has been demonstrated with time‐resolved hyperpolarized ^3^He MRI during breath‐hold.^4^ However, ^3^He has become increasingly scarce and expensive,^5^ motivating a shift towards ^129^Xe MRI for most applications in the lungs.^6^ The aim of this work was to compare ^3^He and ^129^Xe time‐resolved imaging for the detection of delayed and collateral ventilation in patients with severe COPD.

## Materials and Methods

Three patients with advanced COPD under consideration for ELVR were scanned using a 1.5T whole‐body MRI system (GE HDx, Milwaukee, WI) equipped for hyperpolarized gas imaging. This retrospective analysis was conducted with approval of the research governance and ethics board with a waiver of informed consent. Patients 1 and 2 were 64‐year‐old females, patient 3 was a 52‐year‐old male. Patients were positioned supine in a transmit‐receive quadrature vest coil (Clinical MR Solutions, Brookfield, WI) tuned to the appropriate resonance frequency of ^3^He or ^129^Xe. Dynamic time‐series ventilation images were acquired during breath‐hold using a 3D coronal balanced steady‐state free‐precession sequence with full lung coverage (field of view [FOV] = 40–48 cm, slice number = 22–24), in‐plane matrix 64 × 32, 10mm slice thickness, and Cartesian centric phase encoding.

100 mL hyperpolarized ^3^He (∼25% polarization; GE Healthcare, Amersham, UK) and 900 ml N_2_ was inhaled from functional residual capacity (FRC). MR sequence parameters: θ = 8.5°, echo time (TE) = 0.5 msec, repetition time (TR) = 1.6 msec, bandwidth (BW) = 167 kHz, scan duration = 21 seconds, and six dynamic volumes acquired at 0, 4, 7, 11, 15, and 19 seconds.

350 mL hyperpolarized ^129^Xe (129‐enriched [86%], ∼30% polarization) and 650 ml N_2_ was inhaled from FRC. MR sequence parameters: θ = 6.5°, TE = 1.4 msec, TR = 4.5 msec, BW = 16 kHz, scan duration = 23 seconds, and six dynamic volumes acquired at 0, 4, 8, 12, 16, and 20 seconds.

To quantify dynamic changes in global lung ventilation, whole lung ventilated volume (VV) was calculated for each timepoint using automated spatial fuzzy C‐means segmentation, a methodology which is robust to noise.^7^

The free diffusion coefficient (D) and 1D mean free diffusion path length (z_rms_ = √(2DΔt)) after time Δt^8^ were estimated for ^3^He and ^129^Xe within the lungs. A volume of 6.6 L of air, corresponding to the average FRC of the three patients, was used to estimate the experimental in situ gas mixture required for the calculation of D.

Volumetric unenhanced thoracic CT images and pulmonary function test results were also reviewed.

## Results

Centrilobular emphysema and hyperinflation were evident on the CT images of all patients. Patients 1, 2, and 3 had forced expiratory volume in 1 second of 28.5, 24.7, and 27.4 percent predicted, and residual volume of 272.1, 291.5, and 223.3 percent predicted, respectively. Patient 1 lost breath‐hold after 13 seconds of ^3^He data acquisition and after 20 seconds of ^129^Xe data acquisition; patients 2 and 3 performed both breath‐holds successfully.

**Figure 1:**
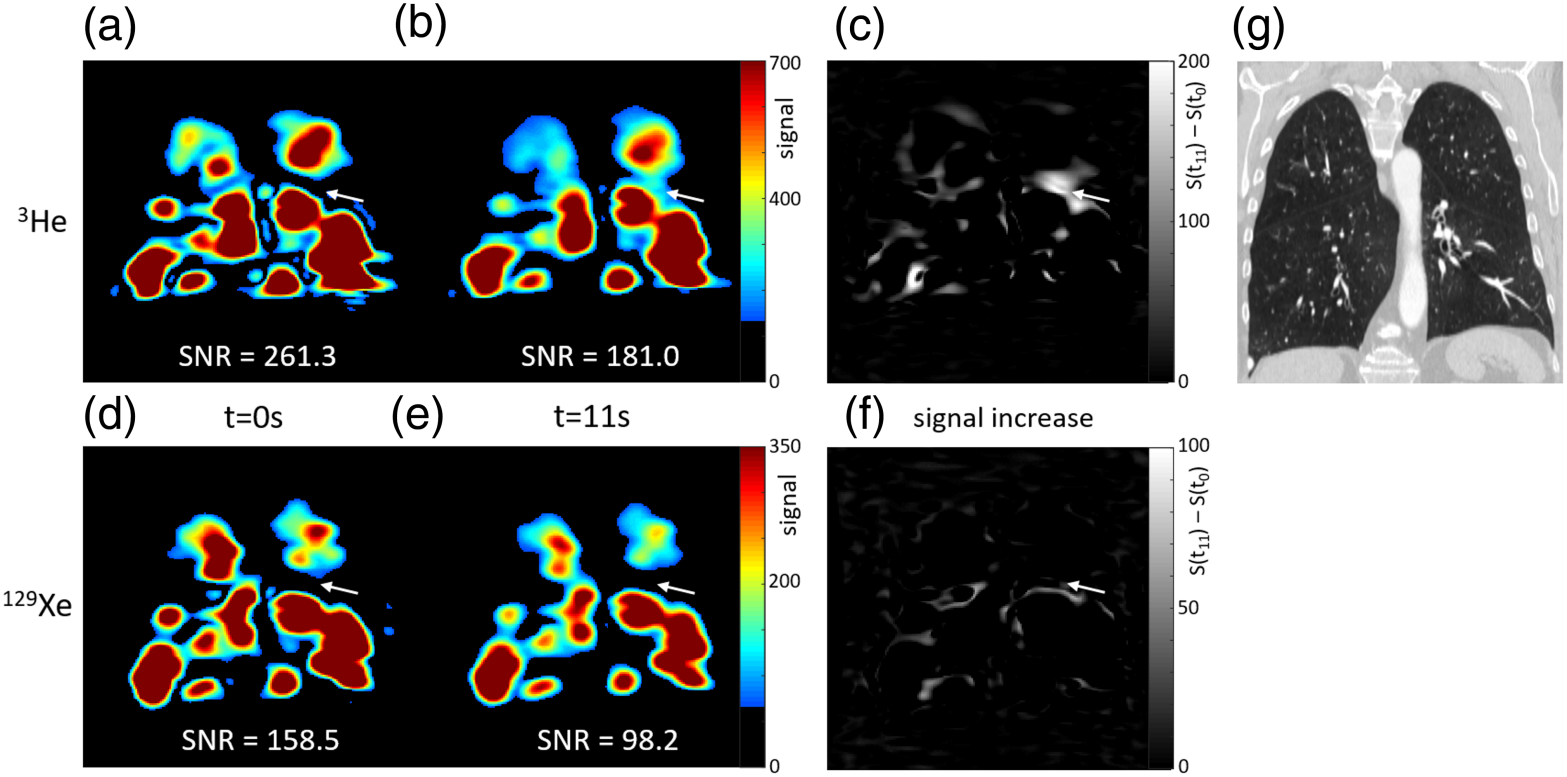
^3^He images (top) and ^129^Xe images (bottom) acquired from patient 1 during breath‐hold; (a,d) at the start of the imaging sequence, t = 0 sec, and (b,e) after 11 seconds, both shown with the same signal range. (c,f) Maps of signal increase from t = 0 sec to t = 11 sec. White arrows highlight a region of lung where ^3^He signal increased over time, but ^129^Xe signal did not. The coronal unenhanced thoracic CT (g) showed moderate centrilobular emphysema and hyperinflation, and an intact left oblique fissure. Mean signal‐to‐noise ratio (SNR) over the whole lung ventilated volume for that timepoint is displayed for a, b, d, and e.

**Figure 2:**
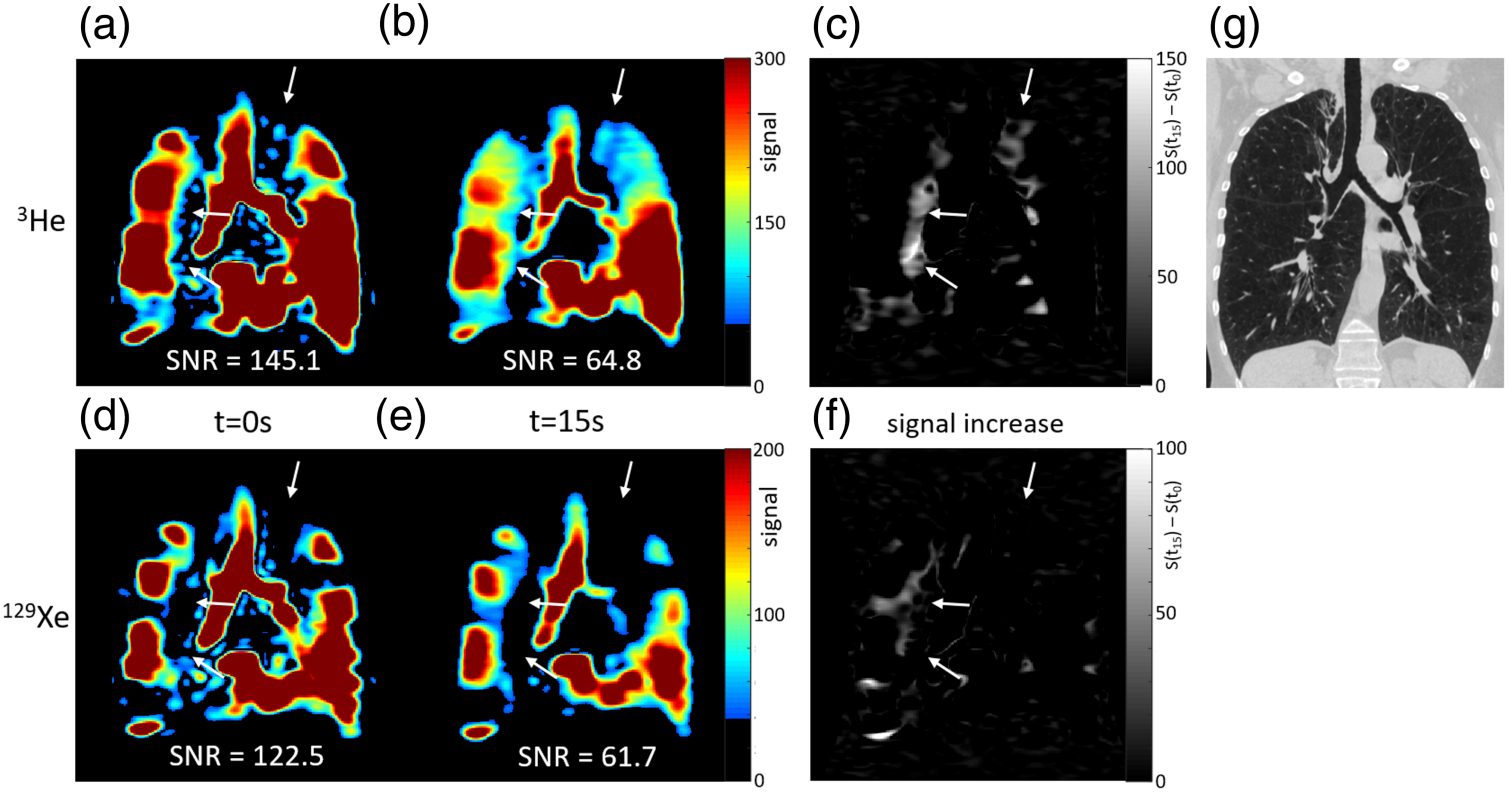
^3^He images (top) and ^129^Xe images (bottom) acquired from patient 2 during breath‐hold; (a,d) at the start of the imaging sequence, t = 0 sec, and (b,e) after 15 seconds, both shown with the same signal range. (c,f) Maps of signal increase from t = 0 sec to t = 15 sec. White arrows highlight regions of lung where ^3^He signal increased over time, but ^129^Xe signal did not. (g) CT showed severe centrilobular emphysema and severe hyperinflation, and all fissures appeared intact. Mean SNR over the whole lung ventilated volume for that timepoint is displayed for a, b, d, and e.

**Figure 3:**
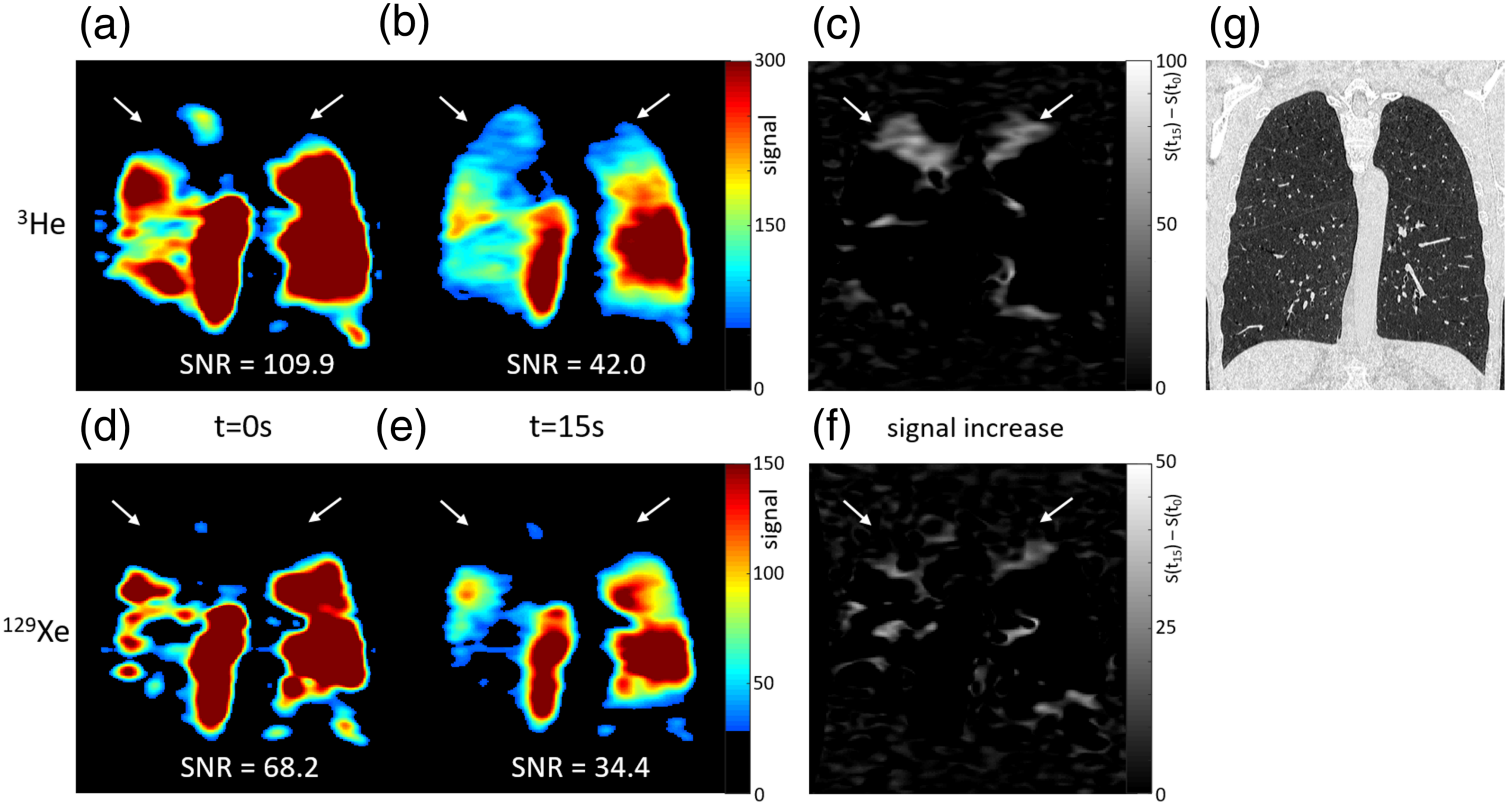
^3^He images (top) and ^129^Xe images (bottom) acquired from patient 3 during breath‐hold; (a,d) at the start of the imaging sequence, t = 0 sec, and (b,e) after 15 seconds, both shown with the same signal range. (c,f) Maps of signal increase from t = 0 sec to t = 15 sec. White arrows highlight regions of lung where ^3^He signal increased over time, but ^129^Xe signal did not. (g) CT showed severe centrilobular emphysema and hyperinflation, and all fissures appeared intact. Mean SNR over the whole lung ventilated volume for that timepoint is displayed for a, b, d, and e.

The figures show ^3^He and ^129^Xe images of gas distribution within the lungs at the first timepoint and a later timepoint during breath‐hold. Arrows highlight initially nonventilated lung regions where signal increased over time in the ^3^He images, but not in the ^129^Xe images. Some evidence of delayed ventilation was observed with ^129^Xe but only within lung regions that were ventilated at t = 0 sec with ^3^He (Figs. 1, 2, 3).

Whole lung ventilated volume increased over time for both gases, but the ratio VV_3He_/VV_129Xe_ was greater at the end of the breath‐holds than at t = 0 sec. VV_3He_/VV_129Xe_ increased from 1.10 to 1.19 for patient 1, from 1.37 to 1.54 for patient 2, and from 1.25 to 1.31 for patient 3.

The ratio of ^129^Xe diffusivity to ^3^He diffusivity within the hyperinflated lungs of a patient with an FRC of 6.6 L was 0.15 for the estimated experimental gas mixtures (D_(129Xe‐air,lungs)_ = 0.13 cm^2^s^‐1^, D_(3He‐air,lungs)_ = 0.87 cm^2^s^‐1^) . This was associated with a mean free diffusion path length of 2.0 cm for ^129^Xe and 5.1 cm for ^3^He on the time‐course of the time‐resolved experiment (Δt = 15 sec).

## Discussion

The visualization of delayed and collateral ventilation with ^3^He but not with ^129^Xe, and the increased VV_3He_/VV_129Xe_ ratio at the end of the breath‐holds compared to t = 0 sec, are likely due to the large difference in diffusivity between the gas mixtures used. The observation of reduced ventilated volume in ^129^Xe images when compared to ^3^He images acquired from the same patients with COPD has been reported before for single timepoint ventilation imaging.^9^ The diffusion coefficient of ^129^Xe diluted in air (0.14 cm^2^s^‐1^)^8^ is closer to that of air alone (0.22 cm^2^s^‐1^)^10^ than ^3^He diluted in air (0.86 cm^2^s^‐1^).^8^ However, the higher diffusivity of ^3^He highlights delayed ventilation which would take place on a longer time‐scale for pure air rather than the ^3^He‐air mixture used for imaging; for example, it would take 60 seconds for pure air to travel the same mean free diffusion path length as the ^3^He‐air mixture within the lungs would travel in 15 seconds. Even if it were feasible to image ^3^He and ^129^Xe at the same mean free diffusion path length, other inherent differences between the two gases, such as increased density and viscosity of ^129^Xe compared to ^3^He, may affect the relative sensitivity of ^3^He and ^129^Xe MRI.

In conclusion, although the number of patients studied was small, all showed instances where delayed and collateral ventilation were detected with ^3^He MRI but not observed using ^129^Xe MRI, indicating a limitation of time‐resolved ^129^Xe MRI for this emergent application.

## Grant Support

This work was funded by the Medical Research Council (MR/M008894/1) and the National Institute of Health Research (NIHR‐RP‐R3‐12‐027). The views expressed in this publication are those of the authors and not necessarily those of the National Health Service, the National Institute for Health Research or the Department of Health.

## Supporting information

Supporting information Figures
