# Supplementary material for: Imaging Collateral Ventilation in Patients With Advanced Chronic Obstructive Pulmonary Disease: Relative Sensitivity of 3He and 129Xe MRI
Source: J Magn Reson Imaging. 2018 Sep 29;49(4):1195–7. doi: 10.1002/jmri.26273 (PMC6749943; doi:10.1002/jmri.26273)
Supplement: Supplementary file 1 — Supporting information Figures [file JMRI-49-1195-s001.doc]

**Supporting Information**

**Figure 1**


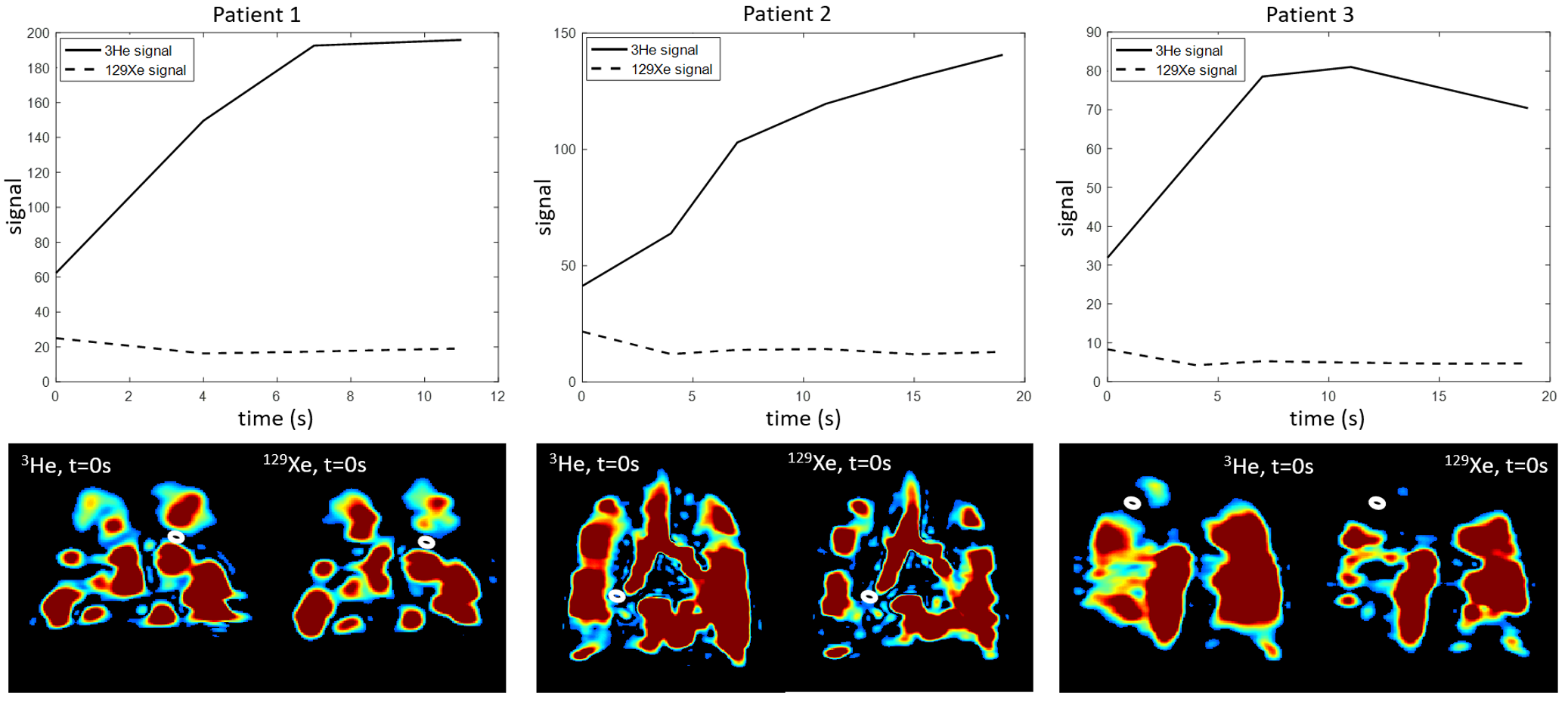


Region of interest plots of signal over time during breath-hold for (left) patient 1, (center) patient 2, and (right) patient 3. ^3^He signal (solid line) increased over time while ^129^Xe signal (dashed line) did not. Images show the placement of the regions of interest (white ovals) on the t=0s images.

**Figure 2**

**
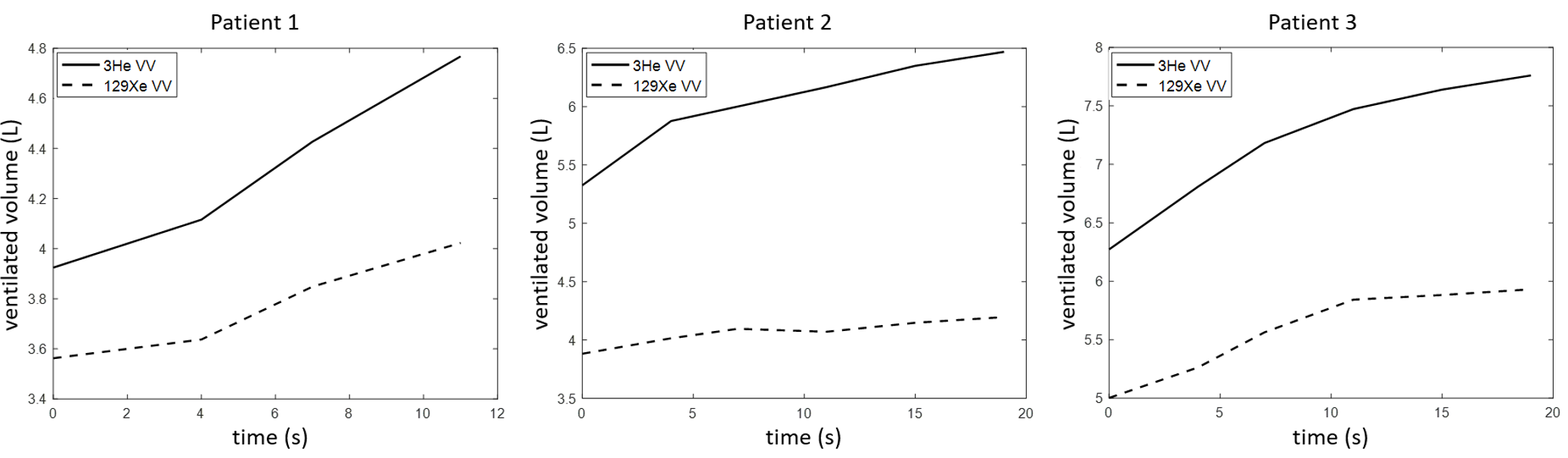
**

Plots of whole lung ventilated volume over time for (left) patient 1, (center) patient 2, and (right) patient 3. ^3^He ventilated volume (solid line) was greater than ^129^Xe ventilated volume (dashed line), and VV increased over time for both gases.
